# Understanding Deaths in Custodial Environments: A Systematic Review of Global Trends and Risk Factors

**DOI:** 10.7759/cureus.112871

**Published:** 2026-07-17

**Authors:** Nikoleta Makri, Nikolaos Kalogrias, Konstantinos Katsos, Dimitrios Vlachodimitropoulos, Ioannis Papoutsis, Chara Spiliopoulou, Emmanouil I Sakelliadis

**Affiliations:** 1 Department of Forensic Medicine and Toxicology, School of Medicine, National and Kapodistrian University of Athens, Athens, GRC; 2 Forensic Service of Athens, Ministry of Justice, Athens, GRC

**Keywords:** death in prison, forensic investigation, inmate mortality, prisoner death, systematic review

## Abstract

Deaths in custodial settings mark a critical intersection of human rights, public health, and state accountability and serve as a key indicator of systemic deficiencies within correctional institutions. Evidence consistently shows that suicide is the leading cause of death in custody, especially during the early detention period. This trend reflects increased psychological vulnerability, pre-existing mental health issues, and limited access to specialized care. Natural causes - such as cardiovascular and respiratory diseases, infections, and complications from chronic illnesses - also make up a significant portion of deaths, often associated with limited healthcare access, delayed treatment, and resource shortages, particularly in overcrowded or underfunded facilities. Additional factors include substance abuse, absence of rehabilitation programs, and violent incidents, suggesting potential limitations in supervision, policy enforcement, and institutional oversight. This study was conducted using a systematic review methodology, with the aim of identifying and synthesizing evidence on the causes of death among individuals in custody across different detention settings and geographical regions. The findings emphasize the ongoing disconnect between international legal frameworks, such as the United Nations Standard Minimum Rules for the Treatment of Prisoners (the Mandela Rules), and their actual application across jurisdictions. As a response, the review offers several evidence-based recommendations: mandatory medical and psychiatric assessments before detention, improved healthcare infrastructure, the adoption of rehabilitation and substance control programs, and the creation of independent investigative and monitoring bodies for all custodial deaths. Ultimately, reducing deaths in custody requires coordinated healthcare, institutional, and monitoring strategies aimed at improving detainee safety and healthcare outcomes. Protecting the lives and dignity of detainees remains an important responsibility of custodial institutions and public health systems.

## Introduction and background

The deaths of detainees constitute a complex and sensitive issue that touches upon human rights, public health, and state responsibility [1]. Ιnternational human rights frameworks emphasize the protection of life and dignity during detention [2]. Nevertheless, mortality statistics within police departments and prisons reveal that this state obligation is not fully met. Deaths in custody, whether due to natural or violent causes, represent an important public health and institutional concern within correctional systems specifically and of state care more broadly [3].

The study of deaths in prison environments holds particular significance and value, as it reveals that this issue remains largely invisible to the broader public [4]. Moreover, several studies suggest that some contributing factors associated with custodial deaths may be identifiable at earlier stages [5,6]. In particular, suicide [7], which is one of the most frequently studied causes, has been shown to be among the leading causes of death in such environments, with the majority occurring within the first hours of detention [8]. This likely happens because detainees are psychologically vulnerable, often with a history of mental illness or substance dependency. Therefore, the lack of specialized mental health personnel, the insufficient training of available staff, and the absence of monitoring and risk-prevention mechanisms are factors that may further increase vulnerability within detention facilities [9].

At the same time, deaths from natural causes also represent a multidimensional issue, especially among inmates with pre-existing health conditions. The most common causes of death, as reported in relevant studies, include cardiovascular disease, respiratory conditions, liver failure, HIV, and infections from the SARS-CoV-2 virus [10-12], which was among the leading causes of death in 2020 and 2021. Limited access to medical services, delayed therapeutic interventions, and a shortage of personnel and resources remain important challenges to ensuring the physical and mental well-being of incarcerated individuals [13]. Naturally, these issues may be more pronounced in developing countries or those facing prison overcrowding, where healthcare infrastructure is minimal or even nonexistent [14-16].

Additionally, another major factor contributing to increased mortality within detention facilities is the abuse of substances and prescribed drugs. The illicit distribution of narcotics within correctional facilities, their uncontrolled usage, and the interaction of substances often result in fatal incidents [17]. The absence of awareness and rehabilitation programs, combined with the lack of a coherent drug control policy, may contribute to increased mortality risk among detainees [18]. Furthermore, violent deaths - whether perpetrated by prison staff or among inmates - continue to be reported globally, raising serious concerns regarding oversight and transparency.

The unique characteristics of the incarcerated population have led to the development of international legal frameworks through bodies such as the United Nations (the Mandela Rules), the World Health Organization, and the European Convention on Human Rights [19,20]. This reflects acknowledgment of the state's responsibility for protecting prisoners. However, it also becomes evident that the implementation of these frameworks varies significantly across countries, with problems more frequent in systems lacking independent monitoring mechanisms or institutional transparency.

The systematic review that follows aims to highlight the patterns, trends, and divergences among studies examining deaths in custodial settings. Specifically, it analyzes data across geographic regions, types of detention, and causes of death to draw reliable, comprehensive conclusions that can inform preventive policy and foster more effective institutional interventions. This endeavor does not seek to offer a mere descriptive overview; rather, it aspires to document in depth the diverse data, highlighting the importance of preventive measures and improved custodial healthcare practices within one of the most marginalized environments: correctional and police detention facilities.

## Review

Methods

This systematic review was designed and organized in accordance with the Preferred Reporting Items for Systematic Reviews and Meta-Analyses (PRISMA) 2020 guidelines to ensure transparency, completeness, and reproducibility throughout the stages of searching, selecting, and synthesizing the available literature [21].

This study was conducted using a systematic review methodology, with the aim of identifying and synthesizing evidence on the causes of death among individuals in custody across different detention settings and geographical regions. The review question was structured according to a Population, Exposure, Outcome (PEO) framework, in which the population comprised incarcerated individuals, the exposure referred to custodial settings and detention conditions, and the outcome concerned the causes and patterns of mortality in custody. The review focused on the collection and analysis of both quantitative and qualitative data derived from published studies that met predefined eligibility and validity criteria. A review protocol tailored to the specific research question was developed and followed throughout the study’s design and implementation.

A comprehensive literature search was conducted in the PubMed and Scopus electronic databases. The search covered publications from 1978 to 2025, reflecting the availability of systematic reporting on custodial deaths in the international literature. A combination of controlled vocabulary and free-text terms was used, including but not limited to: “post-mortem examination”, “deaths in prison”, “custodial deaths”, “custodial mortality”, “inmates”, “detainees”, “inmate death statistics”, “homicide”, “suicide”, “defense wounds”, “stab wounds”, “illness”, “disease”, and “overdose”, along with relevant synonyms and spelling variations. Boolean operators (AND/OR) were applied where appropriate to combine search terms and improve search sensitivity. The search strategy was adapted as necessary, and only studies published in peer-reviewed scientific journals were considered eligible to ensure the scientific quality and credibility of the included evidence. No restrictions were applied to the geographical region.

Studies were included in the review if they met the following criteria: explicit reporting of the cause of death, including cases classified as “undetermined”; availability of demographic data (minimally age and sex); inclusion of at least 10 cases per study, in order to reduce the influence of isolated case reports and improve comparability across studies; clear reference to the type of custodial setting, such as prison, police custody, or psychiatric detention; and sufficient description of the study population and methodological approach. Studies were excluded if they consisted solely of single case reports, opinion articles, commentaries, editorials, or publications lacking primary empirical data. Studies with insufficient sample descriptions or unclear outcome definitions were also excluded.

After removing duplicates, all identified publications were initially screened for title and abstract using the online platform Rayyan (Rayyan Systems, Inc., Cambridge, USA), which was used to organize, index, and systematically evaluate potentially eligible studies [22]. Articles deemed potentially eligible were read in full text.

Study selection and data extraction were conducted independently by two reviewers, and in cases of disagreement or uncertainty, a third reviewer was consulted to reach consensus. For each included study, data were extracted on country of research, sample size, observation period, causes of death, age, and sex of the study population, type and location of detention, and study design. A predefined standardized extraction form was used to ensure consistency during data collection. Formal inter-rater reliability statistics (e.g., kappa statistics) were not calculated, and this is acknowledged as a methodological limitation.

The methodological quality of the included studies was assessed using the Joanna Briggs Institute critical appraisal tools, with the appropriate checklist selected based on each study’s design (e.g., cross-sectional, cohort, case series, or registry-based analyses) [23]. The quality appraisal focused on key methodological aspects, including the clarity of the outcome definition, the adequacy of data sources, the completeness of reporting, and potential sources of bias. The assessment was used to inform the interpretation of findings but did not serve as a basis for excluding studies that otherwise met the inclusion criteria. Due to heterogeneity in study designs and reporting methods, formal quantitative quality stratification was not performed.

Given the substantial heterogeneity in study designs, data sources, outcome definitions, and reporting practices, a quantitative meta-analysis was deemed methodologically inappropriate. Formal subgroup analyses and sensitivity analyses were also not feasible due to variability in study methodology and outcome reporting. Instead, a narrative synthesis approach was employed, allowing for systematic comparison of findings across studies. Quantitative data were summarized descriptively, while qualitative patterns and recurring trends were identified through comparative analysis, with particular emphasis on causes of death, demographic characteristics such as age and sex, type of detention, geographical context, and the timing of death relative to the period of custody.

This systematic review was not prospectively registered in the International Prospective Register of Systematic Reviews (PROSPERO). Although PROSPERO accepts systematic reviews of observational studies, a predefined review protocol was developed and followed throughout the study process. The absence of protocol registration is acknowledged as a limitation affecting transparency and reproducibility. 

Results

**Figure 1:**
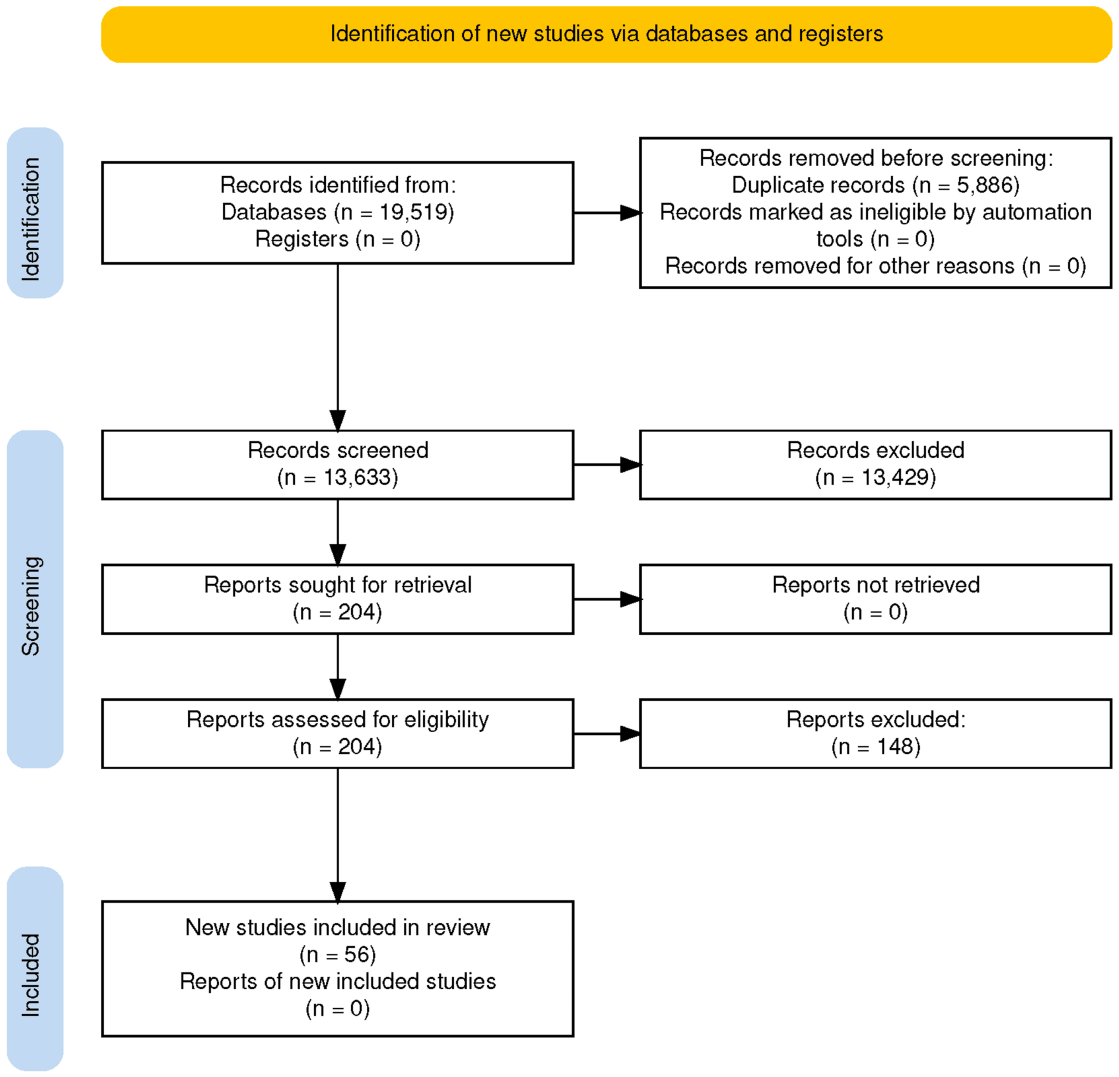
PRISMA flow chart of this review PRISMA: Preferred Reporting Items for Systematic Reviews and Meta-Analyses

A systematic literature search was conducted in the PubMed and Scopus databases, identifying a total of 19,519 records. Following the removal of duplicates (n = 5,886), 13,633 records remained for screening. Titles and abstracts were reviewed, resulting in the exclusion of 13,429 records. The full texts of 204 articles were subsequently assessed for eligibility. Of these, 148 articles were excluded for reasons including insufficient data, unclear outcomes, inappropriate study design, or irrelevant populations. Ultimately, 56 studies fulfilled the inclusion criteria and were included in the systematic review. The study selection process is presented in the PRISMA flow diagram (Figure 1).

The papers included in the systematic review are presented in Table 1.

**Table 1:** Papers included in the systematic review

Author (year)	Country	Title	Primary setting/place of death	Manner of death
Copeland (1984) [24]	USA-Florida	Deaths in custody revisited	Prison (Jail)	Natural
Copeland (1989) [25]	USA-Florida	Fatal suicidal hangings among prisoners in jail	Prison (Jail)	Suicide
Green et al. (1993) [26]	Canada	A study of 133 suicides among Canadian federal prisoners	Prison	Suicide
Joukamaa (1997) [27]	Finland	Prison suicide in Finland, 1969-1992	Prison	Suicide
Joukamaa (1998) [28]	Finland	The mortality of released Finnish prisoners; a 7 year follow-up study of the WATTU project	Prison	Natural
McKee (1998) [29]	USA-South Carolina	Lethal vs nonlethal suicide attempts in jail	Prison (Jail)	Suicide
Perez-Carceles et al. (2001) [30]	Spain	Mortality in maximum security psychiatric hospital patients	Prison	Suicide
Koehler et al. (2003) [31]	USA	Deaths among criminal suspects, law enforcement officers, civilians, and prison inmates	Prison	Natural
Dahle et al. (2005) [32]	Germany	Suicide prevention in penal institutions: validation and optimization of a screening tool for early identification of high-risk inmates in pretrial detention	Prison	Suicide
Roessler et al. (2007) [33]	Austria	Death in correctional facilities: opportunities for automated external defibrillation	Prison	Natural
Cohen (2008) [34]	UK	Safe in our hands?: a study of suicide and self-harm in asylum seekers	Prison	Suicide
Sohail et al. (2010) [35]	Pakistan	Postmortem computed tomography for diagnosis of cause of death in male prisoners	Prison	Natural
Lupu et al. (2012) [36]	Romania	Deaths in custody in Romania. A synthetic study	Prison	Natural
Mirza et al. (2012) [15]	Pakistan	Audit of custodial deaths in Karachi - an autopsy-based study	Prison	Natural
Duthé et al. (2013) [37]	France	Suicide among male prisoners in France: a prospective population-based study	Prison	Suicide
Sakelliadis et al. (2013) [38]	Greece	Forensic investigation of suicide cases in major Greek correctional facilities	Prison	Suicide
Sakelliadis et al. (2013) [39]	Greece	The social profile of victims of suicide in major Greek correctional facilities	Prison	Suicide
Austin et al. (2014) [40]	Australia	Prison suicides in South Australia: 1996-2010	Prison	Suicide
Liao et al. (2014) [41]	China	Investigation of the controversial deaths in incarcerated population in Hunan province of China	Prison	Natural
Gauthier et al. (2015) [13]	Switzerland	Swiss prison suicides between 2000 and 2010	Prison	Suicide
Gherman and Chiroban (2015) [42]	Romania	Causes of death among detainees: a statistical study on the casework of the forensic medicine institute in Cluj-Napoca during the period 2000-2014	Prison	Natural
Jadhao et al. (2015) [43]	India	An overview of custodial deaths in Pune six years retrospective study	Prison (Jail)	Natural
Kucmanic and Gilson (2016) [44]	USA-Ohio	Suicide in jail: a ten-year retrospective study	Prison (Jail)	Suicide
Ünal et al. (2016) [45]	Turkey	Custody and prison deaths autopsied in Istanbul between 2010 and 2012	Prison	Natural
Vohra et al. (2016) [46]	India	Custodial death: a two years prospective study	Prison (Jail)	Natural
Bartoli et al. (2018) [47]	France	Suicide by medication overdose in prison: a study of three cases	Prison	Suicide
Iqtidar et al. (2018) [48]	Ireland	Deaths in custody in the Irish prison service: 5-year retrospective study of drug toxicology and unnatural deaths	Prison	Misadventure
Bardale and Ninal (2019) [49]	India	Hanging in custodial setup: a 10 year analytical study	Prison	Suicide
Chérif El Khal et al. (2019) [50]	France	Deaths in prison custody in Toulouse - autopsy study between 2011 and 2017	Prison	Suicide
Mittal et al. (2019) [51]	India	A two-year prospective study of custodial deaths from Punjab region of India	Prison	Natural
Voulgaris et al. (2019) [18]	Germany	Influence of drugs on prison suicide - a retrospective case study	Prison	Suicide
Chahal et al. (2020) [52]	India	Custodial deaths: an unaddressed issue	Prison	Natural
Kuchewar et al. (2020) [53]	India	Custody-related deaths in Maharashtra state of India - analysis of autopsies performed at a medical teaching institute during the period 2000-2018	Prison	Natural
Wu et al. (2020) [54]	China	Retrospective analysis of 172 cases of custodial deaths in China between 1999 and 2016: forensic experience in China	Prison	Natural
Gentile et al. (2021) [55]	Italy	Mortality in prisons: the experience of the Bureau of Legal Medicine of Milan (Italy) (1993-2017): suicides and natural deaths in prison	Prison	Natural
Mahamad Arif et al. (2021) [56]	Malaysia	A 15-year study of death in custody in Tuanku Ja'afar Hospital Seremban	Prison	Natural
Bardale and Ninal (2022) [14]	India	Analysis of elderly deaths in the custodial set-up: a 10-year retrospective study	Prison	Natural
Belli et al. (2024) [3]	Italy	Deaths in jail: a retrospective analysis of autopsies performed at the Legal Medicine Unit of Pavia (1999-2022)	Prison	Suicide
Johnson (1982) [57]	England	Deaths in police custody in England and Wales	Police custody	Unnatural (Poisoning due to alcohol or drug)
Blaauw et al. (1997) [58]	Netherlands	Deaths and medical attention in police custody	Police custody	Suicide
Pollanen et al. (1998) [59]	Ontario	Unexpected death related to restraint for excited delirium: a retrospective study of deaths in police custody and in the community	Police custody	Delirium
Petschel and Gall (2000) [60]	Melbourne	A profile of deaths in custody in Victoria, 1991-96	Police custody	Accident
Bhana (2003) [61]	South Africa	Custody-related deaths in Durban, South Africa 1998-2000	Police custody	Police action (shootings, assault by police, police dogs)
Best et al. (2004) [62]	England	Drug deaths in police custody: is dual diagnosis a significant factor?	Police custody	Unnatural (drug overdose)
Southall et al. (2008) [63]	USA	Police custody deaths in Maryland, USA: an examination of 45 cases	Police custody	Undetermined
Heide et al. (2009) [64]	Germany	Deaths in German police custody	Police custody	Unnatural
Heide et al. (2010) [65]	Germany	An avoidable death in police custody?	Police custody	Unnatural
Yapo Etté et al. (2012) [66]	Ivory Coast	What about deaths in police custody in Abidjan? A retrospective study over 8 years (2001-2008)	Prison custody	Violent
Bardale and Dixit (2015) [9]	India	Suicide behind bars: a 10-year retrospective study	Police lock-ups	Suicide
Aasebø et al. (2016) [67]	Norway	Can deaths in police cells be prevented? Experience from Norway and death rates in other countries	Police cells	Unnatural (alcohol intoxication)
Pathak et al. (2016) [68]	India	Unnatural deaths in police lockup/prisons of North Maharashtra region: a 15 year retrospective study	Police lock-ups	Suicide
Dutta and Sharma (2019) [69]	India	A prospective study of pattern of custodial deaths of autopsies conducted at SMS Hospital Jaipur during 2017-2018	Police custody	Natural
Nwafor et al. (2021) [16]	Nigeria	Retrospective post mortem study of custodial deaths in Uyo, South-South, Nigeria	Police custody	Natural
Soyemi et al. (2021) [70]	Nigeria	Custodial deaths seen in the Office of the Chief Medical Examiner, Lagos, Nigeria: an 11-year autopsy study	Police custody	Natural
Okoye et al. (1999) [71]	USA-Nebraska	An analysis and report of custodial deaths in Nebraska, USA	Custody	Natural
Grant et al. (2007) [72]	USA	Death in custody: a historical analysis	Custody	Natural

Analysis of data from the 56 studies included in the systematic review revealed significant and recurring patterns of causes of death across different detention systems and geographical contexts [3,9,13-16,18,24-72]. In total, more than 6,000 prisoner deaths were recorded, which were categorized into five main groups: suicides, natural causes, violent deaths, deaths due to substance use, and accidents. Notably, a substantial number of studies reported incomplete or unclear classification of the cause of death, particularly in cases initially recorded as natural causes or accidents, without complete forensic documentation.

Further analysis of demographic characteristics by individual cause of death revealed differences across categories. Suicides and deaths related to substance abuse primarily involved younger prisoners, whereas deaths from natural causes were more frequently recorded among older age groups. At the same time, violent deaths exhibited a wider age distribution, while remaining predominantly male in characteristic. These differences were reported with relative consistency across studies from different geographical contexts.

Most of the included studies were retrospective in design and relied mainly on secondary data sources, such as medical examiner records, official death registries, prison records, and police records. The temporal scope of the data varied considerably between studies, ranging from a few years to several decades. In some cases, the data were national in scope, while in others they were limited to specific detention facilities or geographical regions. In addition, heterogeneity was observed in the presentation of results: some studies provided detailed quantitative data, whereas others were limited to descriptive reporting, which affected the comparability of individual findings.

Overall, 94% of the deceased individuals were male, with women accounting for only 6% of the total sample. Further examination of individual studies demonstrated that male predominance occurred with remarkable consistency across all categories of causes of death. Nearly all studies that provided detailed demographic data reported male proportions exceeding 85%, regardless of the primary cause of death. In contrast, female prisoners were either entirely absent from death records or represented by very small absolute numbers, a pattern observed in both European and non-European studies.

The mean age of deceased prisoners was 37 years, with the highest mortality consistently observed in the 21-45 age group. This age range was repeatedly identified across different causes of death and geographical contexts. Nevertheless, variability between studies was noted, with some reporting a peak in cases between the ages of 25 and 35 years, while others extended the upper age limit to 50 years. Older age groups were predominantly associated with deaths from natural causes, whereas younger prisoners appeared disproportionately more frequently in incidents occurring shortly after the onset of detention.

Most deaths occurred in prisons, although cases were also recorded in police custody [64,67]. Differences were observed according to the type of detention, with deaths in police custody more frequently documented shortly after arrest and predominantly associated with suicides or instances of inappropriate or substandard treatment during the initial phase of detention. In contrast, deaths occurring in prisons were distributed over a longer time and were more often associated with natural causes, substance use, and complications related to chronic diseases.

Significant geographical differences were also observed. European studies predominantly reported suicides as the leading cause of death [47,48], whereas studies from Asia [45,46] and Africa [16] documented higher proportions of deaths due to natural causes and violence. Overall, suicide was identified as the most common cause of death, accounting for 36% of all cases [9]. 

Analysis of suicides in relation to sex and age revealed particularly homogeneous patterns. Almost all suicides involved male prisoners, primarily aged 21-40 years. This age distribution was consistently observed across studies from different continents, irrespective of sample size or type of detention. Hanging was the most frequently reported method, typically involving improvised means such as sheets, clothing, or shoelaces [25].

More than half of all suicides occurred within the first 30 days of detention, with several cases documented within the first 72 hours [25,44]. In studies that provided detailed temporal data, suicides among younger detainees were disproportionately more frequent during the initial days or weeks of detention, with no observed differences in suicide methods between age groups.

In studies that reported the duration of detention, the timing of death varied by cause. Suicides and violent deaths occurred more frequently during the early stages of detention, whereas deaths from natural causes and substance use were more commonly associated with prolonged or repeated periods of detention. This pattern was observed across different correctional systems and represented a recurring feature among studies reporting relevant temporal data [29].

The second largest category comprised deaths from natural causes, most commonly cardiovascular disease, infections, and complications from chronic diseases such as hepatitis, renal failure, and chronic pulmonary disease [42]. These deaths were primarily recorded among prisoners aged over 40 years [14].

Violent deaths included assaults by fellow inmates, excessive use of force by security personnel, and cases of torture. Studies conducted outside Europe, particularly in India and Nigeria [16,53], reported instances in which deaths initially classified as natural were later determined, following forensic investigation, to have resulted from violent causes.

Deaths related to substance abuse were recorded in nearly all geographical regions, with increased frequency in countries reporting high rates of drug dependence among incarcerated populations [18]. Several studies from the USA and Ireland [48,63,71] described repeated incidents involving the administration and monitoring of pharmaceutical substances in detention facilities.

Finally, deaths attributed to accidents constituted the smallest proportion and included incidents such as falls, unintentional self-harm, and misuse of sedatives [54]. These cases were most frequently reported in psychiatric institutions or among prisoners with severe mental disorders or intellectual disabilities.

Overall, the findings indicate that sex and age represent consistent demographic factors in deaths occurring within detention settings. The repeated predominance of male prisoners and the concentration of deaths among young and middle-aged individuals were observed with notable consistency, particularly in cases of suicide [37], irrespective of geographical context or type of detention. Additionally, several studies identified differences in death recording and classification practices across correctional systems, resulting in variability in the availability of comparable data between countries and time periods.

Discussion

The analysis of causes of death highlights numerous recurring patterns and institutional challenges in detention settings, which persist regardless of geographical region or time [24,70]. High suicide rates, inadequate medical care, deaths from substance abuse, and violent incidents - such as assaults between inmates, excessive use of force by custodial staff, and, in some cases, ill-treatment during detention - are not isolated events [16]. Rather, they highlight persistent institutional and healthcare challenges affecting incarcerated populations [3,31,72].

The increasing number of suicides in prisons, especially during the early days of detention, underscores the lack of psychiatric evaluation and monitoring during the most critical period requiring support for adaptation to a highly stressful environment [37]. This limitation may reduce the opportunity for timely intervention during periods of acute psychological distress [26]. The high number of deaths from natural causes may reflect limitations in healthcare access and availability within custodial settings [52]. While prisons are not expected to cure all illnesses, it remains important to identify and respond to medical needs [28]. Many inmates enter custody with already poor health histories, and a lack of access to at least basic medical care may contribute to adverse outcomes. Delays in addressing urgent medical situations and understaffing of qualified personnel further compound this issue. The findings may indicate limitations in preventive healthcare practices within custodial settings and gaps in custodial healthcare practices and institutional preparedness [45].

Deaths attributable to substance abuse indicate important challenges regarding the effectiveness of security and oversight mechanisms, highlighting important institutional and healthcare challenges related to detainee safety [62]. The lack of organized rehabilitation programs and the disconnection of detention facilities from public health infrastructure may contribute to ongoing patterns of addiction and substance-related harm [73]. In countries like the USA, substance-related deaths have dramatically increased, highlighting the importance of preventive and rehabilitative action [72]. In the USA, substance-related deaths in custodial settings began to be reported more consistently from the late 1980s onward, with a further increase documented during the 1990s and early 2000s [72]. Historical and state-level analyses indicate that these deaths frequently involved acute drug intoxication, particularly cocaine, and occurred during arrest, police custody, or the early stages of detention. This temporal pattern reflects a shift in the epidemiology of custodial deaths and highlights the growing impact of substance abuse on mortality under custody [63,72].

Deaths associated with mistreatment or use of force remain an important concern, often characterized by limited transparency, lack of independent investigation, and challenges in institutional accountability [74]. The absence of mandatory forensic investigation in many countries represents a significant institutional gap that may reduce transparency and institutional accountability in prison systems [68]. The existence of independent oversight mechanisms could serve as institutional safeguards, though their functionality is frequently limited and underdeveloped.

Regarding the geographical analysis of deaths in custody, significant variations were identified in both causes and the relative proportions of each cause across regions. More specifically, it was demonstrated that Asia [15,54], Africa [16,61], Europe [2], and North America [44,72] display distinct patterns and trends, highlighting the importance of context-specific interventions that take into account the specificities of the local context. Conversely, adopting a more optimistic lens, the fact that each region exhibits different distributions of causes of death may serve as a useful tool for designing targeted measures to reduce mortality rates, informed by regional reductions in specific causes.

More precisely, suicides were reported more frequently in Europe [38], particularly in countries of Central and Northern Europe such as Finland [28], France [50], Germany [32], and Switzerland [13]. This finding may be attributed to the prevalent use of pre-trial detention, the intense psychological pressure resulting from stringent incarceration conditions, and prison overcrowding. It is also plausible, however, that the elevated rates in Europe are partly due to more transparent and systematic reporting mechanisms [55]. It is worth noting that certain countries, such as Sweden, have implemented specific suicide prevention programs, which have yielded positive outcomes, as evidenced by a decrease in suicide-related deaths [13]. Nevertheless, despite the existence of progressive institutional frameworks, initial psychiatric assessments are often lacking or entirely absent, and supervisory mechanisms remain ineffective-issues which have been subject to considerable criticism [36].

In North America, particularly in the USA and Canada, further disparities in correctional policies have been observed. Some institutions have adopted pioneering protocols for monitoring inmates exhibiting suicidal ideation, whereas others fail to uphold even the basic standards for investigating and recording deaths [63]. Moreover, deaths attributed to state violence [72] or neglect remain alarmingly prevalent [44], especially when compared to European figures.

In the case of Asia, deaths from natural causes constitute the most prevalent category, particularly in India [53], China [54], and Pakistan [15]. This is to some extent expected, given the high prison populations in these countries, which often result in inadequate healthcare provision and failure to maintain even basic hygiene standards. Infectious diseases, poor nutrition, and delays in the delivery of medical care contribute to a sharp rise in deaths from natural causes. Once again, systematic deficiencies in death documentation and the lack of forensic investigation further complicate this already complex issue.

Nonetheless, the findings from several African studies indicate substantial healthcare and reporting challenges, where the majority of deaths are attributed either to state violence [66] or to infectious diseases [61], such as tuberculosis and AIDS. In countries such as South Africa, Ivory Coast, and Nigeria, there are consistent reports of unsuitable detention facilities, instances of torture [61], and limitations in death reporting and documentation practices, including deaths classified without adequate medical evaluation, fatalities lacking forensic investigation [70], and cases of suspected ill-treatment or infection-related deaths that remain undocumented or ambiguously attributed [66].

Regarding age distribution and considering that age constitutes a critical factor in the study of causes of death among incarcerated individuals - as does gender - important conclusions can be drawn. More specifically, the systematic review of 56 studies revealed that most deceased individuals were male, with proportions exceeding 90% in most of the reviewed works [15,30,38,64]. This finding holds dual significance: first, it effectively reflects the nature of the penal system, which disproportionately incarcerates men; and second, it starkly underscores the relative paucity of research on female criminality. Indeed, such research remains at a preliminary stage globally, often conducted merely for comparative purposes vis-à-vis studies on male prison populations.

Nevertheless, this also suggests that men are more frequently exposed to risks during their time in custody [69]. The male predominance is not only quantitative but also qualitative in nature, as they are often attributable to violence, suicide, or drug intoxication [14]. In contrast, deaths among female prisoners more frequently stem from natural causes or mental health-related issues, such as anxiety disorder and depression [46,69]. Female detainees represent a particularly vulnerable group, often having experienced domestic abuse and pre-existing psychiatric disorders [75,76]. Despite their low representation in custodial settings, deaths among women carry substantial significance in highlighting the urgent need for gender-sensitive institutional measures.

In terms of age, most deaths were concentrated within the 21-45 age group [47,59]. Younger detainees, particularly those between 18 and 30 years old, exhibit a heightened risk of suicidal ideation - especially during the initial period of incarceration [9,26,44]. This increased vulnerability may be associated with the impulsivity and recklessness typical of youth, the abrupt severance of social ties, the psychological shock of imprisonment, and the concurrent prevalence of substance use in this demographic.

Conversely, among inmates over the age of 50, natural causes were more frequently identified as the primary cause of death, with cardiovascular diseases, malignancies, and infections being the most common [33]. The lack of preventive screening programs and the absence of age-specific care initiatives contribute significantly to the elevated mortality in this group, compounded by insufficient staffing, poor training, and delayed response in crisis [31]. Furthermore, several studies reported notable delays in transferring elderly inmates to healthcare facilities and in administering appropriate pharmacological treatment [52].

In conclusion, based on the analysis, the causes of death in custody are not merely medical events but social phenomena, intimately linked to the inmate's position within the system. Poverty, physical and mental illness, addiction, and marginalization significantly influence outcomes during incarceration [34]. The study of deaths in custody offers a reflection of how society treats its vulnerable citizens, revealing systemic flaws-but also, in some cases, areas of progress.

Limitations 

As is often the case with systematic reviews, the present study is subject to certain limitations that affect the interpretation of its findings. Most notably, a significant limitation is the heterogeneity among the primary studies included. Disparities in methodologies, inclusion criteria, and case definitions posed considerable challenges to comparison and statistical integration. Furthermore, the varied approaches to the issue of prisoner deaths across different countries resulted in incomplete documentation of key variables-such as length of incarceration, precise age, psychiatric condition, and medical history in cases of natural deaths - thus hindering in-depth analysis of the underlying causes.

Additionally, geographical imbalances were observed in the distribution of the available data. Studies from Europe, North America, and parts of Asia - particularly South and East Asia - were disproportionately represented, whereas regions such as Latin America, the Middle East, and large parts of Africa were underrepresented or nearly absent [13,63]. This pattern likely reflects disparities in research capacity, forensic infrastructure, and publication practices rather than true differences in custodial mortality across regions [37,54]. Moreover, differences in institutional transparency across regions may affect the accuracy of custodial death reporting, particularly where institutional accountability is limited, thereby increasing the risk of publication bias, as severe or sensitive cases may remain unpublished, selectively reported, or ambiguously classified, resulting in censored and incomplete conclusions. Only peer-reviewed studies were included in order to ensure methodological consistency and scientific reliability of the included evidence. However, the absence of grey literature searching may have limited the comprehensiveness of the review. Additionally, the absence of protocol preregistration, limited database coverage, and inability to perform quantitative synthesis may have affected the reproducibility of the findings.

## Conclusions

Nevertheless, despite the limitations, the current review succeeds in identifying robust trends and recurrent patterns, offering meaningful insights into the causes of death in custodial settings. Notably, suicide once again emerges as the leading cause of death among incarcerated individuals, highlighting the importance of immediate preventive measures aimed at reducing its prevalence. Additionally, natural causes of death remain disproportionately high, particularly among elderly prisoners and those with significant preexisting conditions, highlighting the necessity of improving the provision of medical care within detention facilities. Deaths resulting from violence, substance abuse, and accidents further emphasize the need for more effective supervision and enhanced security protocols to prevent such incidents.

These findings may support several evidence-informed considerations for custodial healthcare and monitoring practices. First, it is essential to institutionalize mandatory medical and psychiatric evaluations prior to incarceration, conducted by experienced healthcare professionals with thorough and detailed documentation, to ensure timely access in case of symptom emergence. The development of rehabilitation programs within prisons is equally critical, both to facilitate eventual reintegration and to foster a safer environment for inmates. Furthermore, strengthening forensic independence and mandating investigations into all custodial deaths may be the most crucial step toward meaningful case assessment and effective future prevention. Lastly, adopting international monitoring standards and establishing independent review bodies-consistent with international conventions-would serve to bolster accountability and transparency. Ultimately, the prevention of deaths in custodial settings requires coordinated institutional, healthcare, and monitoring approaches. Further research and improved reporting practices may contribute to a better understanding and prevention of deaths in custodial settings.
